# CBCT-based online adaptive radiotherapy of the bladder – geometrical and dosimetrical considerations compared to conventional IGRT

**DOI:** 10.1186/s13014-025-02710-y

**Published:** 2025-08-14

**Authors:** Jann Fischer, Laura Anna Fischer, Jona Bensberg, Natalia Bojko, Mohamed Bouabdallaoui, Jasper Frohn, Petra Hüttenrauch, Mandy Klingebiel, Daniela Schmitt, Katharina Tegeler, Daniela Wagner, Alina Wenzel, Jessica Moldauer, Niklas Christian Scheele, Hanne Elisabeth Ammon, Stephanie Bendrich, Sandra Donath, Leif Hendrik Dröge, Manuel Guhlich, Andrea Hille, Olga Knaus, Martin Leu, Jan Oelmann, Rami El Shafie, Georg Stamm, Arndt F. Schilling, Stefan Rieken

**Affiliations:** 1 Department of Radiotherapy and Radiation Oncology, University Medical Centre Göttingen, Göttingen, Germany; 2 Comprehensive Cancer Centre, University Medical Centre Göttingen, Göttingen, Germany; 3 Institute for Diagnostic and Interventional Radiology, University Medical Centre Göttingen, Göttingen, Germany; 4 Department of Trauma Surgery, Orthopaedics and Plastic Surgery, University Medical Centre Göttingen, Göttingen, Germany

**Keywords:** Online adaptive radiotherapy, CBCT, Image-guided radiotherapy, Bladder cancer, Target coverage, Organs at risk, Target surface dose, Mercator projection

## Abstract

**Background:**

Bladder cancer radiotherapy presents unique challenges due to the dynamic anatomy of the bladder and the surrounding organs. Conventional image-guided radiotherapy (IGRT) relies on fixed treatment margins and daily couch corrections, which can result in suboptimal dose delivery. Cone Beam Computed Tomography (CBCT)-based online adaptive radiotherapy (oART) allows daily re-optimization of treatment plans, potentially improving target dose coverage while minimizing exposure to organs at risk (OAR). This study compares oART with IGRT in bladder cancer patients.

**Methods:**

160 oART fractions delivered using the Ethos system (Varian Medical Systems, Palo Alto, CA, USA) were analyzed and compared to conventional IGRT. For each adaptive fraction (fx), three plans were evaluated: the scheduled plan (initial plan recalculated based on daily CBCT), the adapted plan (re-optimized to daily anatomy), and the verification plan (dose distribution recalculated on the verification CBCT - vCBCT). Geometric variations, dose-volume parameters and treatment times were analyzed. Clinical plan acceptability was assessed using predefined dose-volume parameters. Dose coverage on the target’s surface was analyzed using a novel method and visualized via Mercator projections.

**Results:**

Despite drinking guidelines, bladder volumes varied significantly day-to-day. Dose coverage of the clinical target volume (CTV) improved significantly with adaptation (median D_98%_ 88.4–97.8%, *p* < 0.01) and further after vCBCT (median D_98%_ 98.1%, *p* < 0.01), with a reduced interquartile range (IQR). Planning target volume (PTV) D_98%_ also improved with adaptation (median 69.5–92.8%, *p* < 0.01) and after vCBCT (median 91.8%, *p* < 0.01), with decreasing IQR. OAR doses showed reduced variability and a measurable dosimetrical benefit. Spatial dose distribution on the surface of the targets improved for adaptation. Plan acceptability in retrospect almost doubled from 11.9% for scheduled plans to 23.1% for adapted plans and 22.5% for verification plans. The scheduled plans were never chosen for treatment. Median oART treatment time was 14 min, compared to 9 min for IGRT.

**Conclusions:**

Treatment times were approximately 1.5 times longer than IGRT; however, CBCT-based oART enhanced target dose coverage, reduced OAR doses, and decreased variability in both target and OAR doses compared to IGRT, while also improving plan acceptability, although the results should be interpreted with caution due to the limited sample size and single-center design.

**Trial registration:**

Not applicable.

**Supplementary Information:**

The online version contains supplementary material available at 10.1186/s13014-025-02710-y.

## Background

Bladder cancer ranks as the ninth most commonly diagnosed cancer globally [1]. Radiotherapy serves as a widely utilized treatment modality for affected patients [2]. Radiotherapy of the bladder presents unique challenges due to the dynamic nature of the target volume and shape as well as surrounding organs at risk (OAR), which can also undergo significant volumetric and positional changes during treatment [3]. Conventional image-guided radiotherapy (IGRT) relies on pre-planned treatment strategies with fixed margins, along with daily translational and possibly rotational couch corrections to address these variations [4]. This is often combined with eating, drinking or voiding instructions, although their benefits remain unpredictable [5, 6]. Various bladder management devices, such as ultrasound-based monitoring systems [7, 8] or bladder catheters [9], have also been explored to achieve reproducible bladder filling. However, these methods are often labor-intensive, invasive or uncomfortable for the patient, and potentially unreliable in terms of reproducibility. Their use may be considered in cases where online adaptive radiotherapy is not available. Currently, margins are typically derived from patient population statistics rather than tailored to individual patient anatomy. This may lead to suboptimal dose delivery, either by underdosing the clinical target volume (CTV) or by overexposing nearby volatile OAR, such as the rectum and bowel.

Potential approaches to address this issue include the use of a library of plans [10, 11] or daily re-optimization of an initial treatment plan, with the latter demonstrating superiority in terms of dose distribution [12, 13]. Cone-Beam Computed Tomography (CBCT)-based [14], as well as Magnetic Resonance Imaging (MRI)-based online adaptive radiotherapy (oART) are promising solutions as they enable daily re-optimization of treatment plans based on the patient’s current anatomy as observed in real-time imaging [15–18]. This approach has already demonstrated improved target dose coverage and reduced variability in dose exposure to OARs in other anatomical regions, such as the vulva [19], prostate bed [20], liver [21], lung [22], head and neck [23] and female breast [24]. Preliminary evidence suggests oART may offer advantages over conventional IGRT also in bladder cancer [25, 26], even though long-term results confirming a clinical benefit of oART are still lacking.

In this study, we present a clinical evaluation of oART for bladder cancer using the Ethos system (Varian Medical Systems, Palo Alto, CA, USA), focusing on its impact on target coverage, OAR sparing, and workflow efficiency. We measured key metrics, including dose-volume parameters, target surface dose, geometric variations, and treatment times and compared them to conventional IGRT, based on data from non-adaptive bladder cancer treatments conducted within our own department.

## Materials, patients and methods

### Study design and patient selection

The Ethos system (version 1.1) is a linear accelerator with a ring-gantry design that features Artificial-Intelligence (AI)-driven kV-CBCT-based oART capabilities. Installed at the University Medical Centre Göttingen in 2022, the Ethos system was used for the first 160 oART sessions analyzed in this study, administered to eight consecutive patients undergoing adaptive bladder cancer treatment. All of them were treated between 2022 and 2024. The allocation of patients to this study was mainly dictated by the availability of treatment slots at the Ethos system. In addition, patients had to provide informed consent and be considered clinically capable of tolerating the longer treatment session time associated with the adaptive workflow. For comparison, 10 patients receiving conventional IGRT bladder treatment during that period were included in the analysis. These comparison cases were also treated using the Ethos system but without adaptation, applying standard image-guidance workflow. Each patient in this study received a total dose of 55 Gy, delivered in 20 fractions to the bladder (CTV), with an additional 5 mm margin (planning target volume, PTV), minus potential bowel loops. The 5 mm margin was composed of 1 mm gantry-collimator inaccuracy, 1 mm couch positioning inaccuracy, and 3 mm to account for potential intrafractional micromovements of the patient. This analysis was approved by the local ethics committee (approval number 7/8/23) and conducted in accordance with the Declaration of Helsinki.

### Workflow description

A comprehensive description of the Ethos adaptive workflow was provided in a previous study [20]. Before treatment, an initial radiation treatment plan was created for each patient using planning computed tomography (pCT) as the primary imaging modality, supplemented by additional imaging if clinically necessary. During each treatment session, a CBCT was acquired, and the pCT was deformably registered to it, generating a synthetic CT (sCT) that reflected the anatomy of the day while preserving the Hounsfield units (HU) from the pCT. This sCT was hidden from the operator and was solely used for dose calculation. OAR and the CTV were automatically delineated on the current CBCT using AI, allowing the clinician to make modifications. The creation of the sCT as well as the AI-based contouring of OAR and target were implemented by the vendor and are certified for clinical use across all installations of the system. They require no user-side customization or configuration. Once all structures were approved, the system calculated two plans: the scheduled plan (SCH), which is the recalculated initial plan based on the day’s anatomy, and the adapted plan (ADP), which is a re-optimized treatment plan. For ADP, the original beam geometry in terms of gantry and collimator angles was maintained, while the multileaf collimator (MLC) shapes were re-optimized and the corresponding number of monitor units (MU) were automatically determined accordingly. The optimization objectives used for re-optimization were inherited unchangeable from the initial radiation treatment plan. After the clinician selected one of these plans, another CBCT (verification CBCT, vCBCT) was taken to account for any potential movement during the adaptation process up to that point, with corresponding translational couch adjustments made as needed. Subsequently, the patient was treated according to the selected plan and an automatic dose reconstruction of the chosen plan on the vCBCT (verification plan - VER) was performed by the system.

### Plan generation, endpoints and statistics

All patients underwent an initial treatment planning procedure adhering to the local standard clinical protocol. This included a pCT scan using a Philips Brilliance Big Bore with 3 mm slice thickness. Patients were instructed to have an emptied bladder for both the pCT and daily radiotherapy sessions. To facilitate this, they received written guidelines on appropriate drinking and urination behaviors. Table 1 outlines the dose-volume specifications for both target volumes and OAR used during the initial treatment planning. Common rectal dose constraints from literature (e.g [27]). were consistently undercut, resulting in low rectal doses in all cases. In line with this, rectal exposure was further reduced following the ALARA-principle (“As Low As Reasonably Achievable”).

**Table 1:** Recorded Dose-Volume-Histogram (DVH) parameter for target volumes and organs at risk. ALARA: as low as reasonably achievable

Organ	Dose-Volume-Specification	Clinical Standard
CTV	D_98%_	≥ 95%
PTV	D_98%_	≥ 95%
PTV	D_2%_	≤ 105%
Rectum	D_mean_	ALARA
Bowel	D_mean_	ALARA
Bowel	D_0.1 cc_	< 50 Gy

Several patients showed anatomical variations, leading to partial overlap between bowel loops and the PTV. To meet the D_0.1cc_ dose specification for the bowel listed in Table 1, the relationship between CTV and PTV was defined as follows:

$${\rm{PTV = }}\left({{\rm{CTV + 5mm}}} \right){\rm{ - Bowel}}$$

This adjustment ensured that, in the presence of nearby bowel loops, the PTV was cropped in the overlapping region while still fully covering the CTV. In these cases, treatment planning prioritized bowel sparing, and consequently any necessary underdosage to the PTV or even CTV was accepted to ensure that bowel dose constraint D_0.1cc_ < 50 Gy was not exceeded. Based on clinical experience, this approach typically results in > 95% coverage of the CTV and > 90% coverage of the (partially cropped) PTV, which was considered clinically sufficient.

All patients received either 9- or 12-field equally spaced IMRT (Intensity Modulated Radiotherapy), as VMAT (Volumetric Modulated Arc Therapy) was not used for adaptation due to the considerably longer time required for calculation and optimization. For each treatment session, the following parameters were documented: monitor units (MU), all dose-volume-histogram (DVH) parameters from Table 1 as well as the CTV volumes. These volumes as well as MU were normalized to the parameter in the initial treatment plan to account for patient-specific interfractional variations. The time between the initial CBCT and vCBCT for each fraction was also recorded. For comparison, the treatment times from a representative sample of 7 sessions for all patients who received conventional IGRT-based radiotherapy for bladder cancer during the study period (10 patients, 70 analyzed fractions in total) were also evaluated (time from start of initial CBCT to end of beam-delivery).

For all fractions, the plan acceptability of SCH, ADP and VER was retrospectively assessed based on clinical standards for CTV and PTV (as specified in Table 1), with D_98%_ values above 95% deemed acceptable. The DICOM structure files of the CTV from each treatment session were compared to those of the initial plan, and the Jaccard index of the bladder (CTV) was calculated as a measure of geometric similarity. The Jaccard index J, also known as Intersection over Union (IoU), for two quantities A and B is defined as:

$$\:J\left(A,B\right)=\:\frac{\left|A\cap\:B\right|}{\left|A\cup\:B\right|}$$

It ranges from zero (no intersection between A and B) to one (perfect overlap).

Comparative statistical analyses on doses and volumes of targets or organs were performed using the Wilcoxon signed-rank test, with patients serving as their own controls. The null hypothesis posited no difference between the parameters of the compared plans, with a two-sided significance level set at *p* < 0.05. Comparative statistical analyses on the acceptability of fractions were performed using the McNemar test, with the null hypothesis stating no difference in acceptability and a significance level of *p* < 0.05. Odds ratios (OR) and corresponding 95% confidence intervals (CI) were calculated to quantify effect size. All statistical analyses were conducted using Python (v3.12) with the packages pandas (v2.2.1), pydicom (v2.4.4), and scipy (v1.12).

### Spatial analysis of dose coverage

**Fig. 1:**
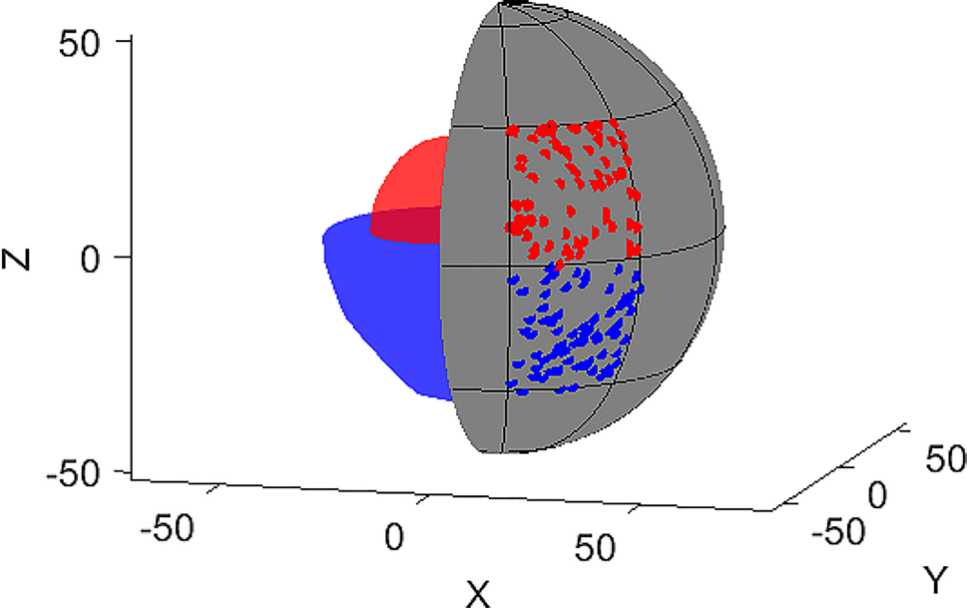
Graphical illustration of spatial analysis of dose coverage: On the left, two partial DICOM CTV (bladder) structures with notably different volumes and shapes (red and blue) are shown. On the right, the partial enveloping unit sphere is displayed, segmented into 5 latitude bands and 10 longitude sectors. Surface points from both the red and blue structures are projected onto two exemplary segments of the sphere for exemplary visualization

The bladder is primarily filled with urine, but only the bladder wall consists of tissue that could meaningfully be targeted by radiation therapy. Common DVH parameters used to describe dose coverage (as D_98%_ or V_95%_) consider the entire organ volume, including its content. To address this limitation, a novel method was applied to specifically assess the dose coverage of the bladder’s surface. For each adaptive treatment session, the SCH and ADP plans were analyzed by extracting structure and dose information from the corresponding DICOM files. The spatial distribution of dose coverage on the surface of the CTV was then assessed. A dense grid of surface points was extracted from the CTV contour, and each point was evaluated to determine whether the local dose met or exceeded 90% of the prescribed dose (49.5 Gy in total or 2.475 Gy per fraction). The 90% threshold was chosen as it corresponds to a typical clinically acceptable D_98%_ dose coverage in cases where bowel constraints necessitate PTV cropping. To enable comparability and presentability even for structures of very diverse volume and shape, these points were projected onto a unit sphere centered at the structure’s center of mass. The sphere was divided into 5 latitude bands and 10 longitude sectors, similar to Earth’s geographic coordinate system, creating 50 segments. This process is illustrated graphically in Fig. 1. To ensure that all points on the surface of the sphere could be comprehensively visualized, the results section employed a Mercator projection of the surface - a conformal cylindrical map projection that represents the spherical data as a rectangular map by preserving angles while distorting areas, particularly near the poles. Mercator projections are widely known and commonly used in cartography, particularly in geographic maps, making them a familiar tool for data visualization [28]. This approach enabled a clear and especially complete presentation of the spatial distribution of data. For each segment, the percentage of points below the dose threshold (failed points) was calculated. The arithmetic mean of failed points per segment and the segment-wise differences in failed point’s ratio between SCH and ADP plans were computed across all 160 fractions.

## Results

### Patient selection

Eight consecutive patients who underwent adaptive bladder irradiation were included in this analysis. Of these patients, seven were male and one was female, with a median age of 72 years [1st quartile (Q1): 68; 3rd quartile (Q3): 83]. A total of 160 fractions were analyzed, all of which were delivered using the adapted plan. The scheduled plan was never chosen for treatment due to inferior dose distribution to the targets and surrounding organs. For ADP, MU did not change significantly compared to the initial plan, with a median relative MU of 0.92 [0.83; 1.09]. To compare treatment times, 70 fractions from 10 bladder cancer patients treated with conventional IGRT during the same time period were evaluated.

### Geometrical differences

**Fig. 2:**
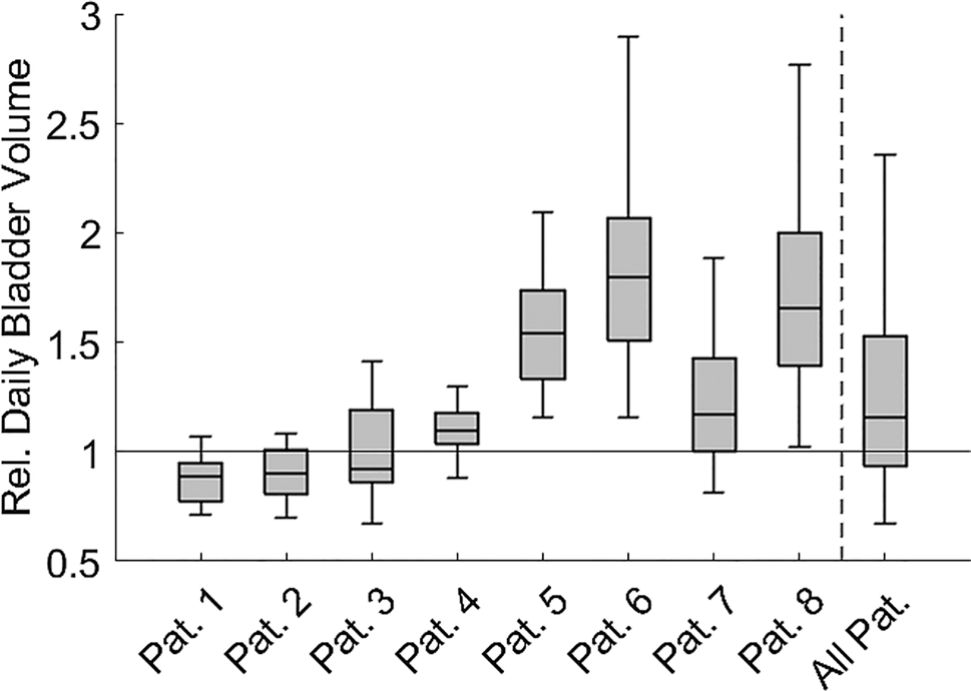
Relative volume of the bladder (CTV) normalized to the initial volume in the pCT, for every individual patient as well as all patients merged

The relative volume of the bladder, normalized to the volume in the pCT, varied across all patients during oART. The median relative volume was 1.16 [Q1: 0.94; Q3: 1.53]. Among individual patients, the median relative volume ranged from 0.89 (patient 1) to 1.80 (patient 6). Figure 2 visualizes these data, with the boxes representing the median, first quartile, and third quartile, while the whiskers extend from the interquartile range (IQR) to the furthest data point not considered an outlier. Data points exceeding 1.5 times the IQR from the edge of the box are considered outliers. This definition is applied to all box plots presented in this work.

**Fig. 3:**
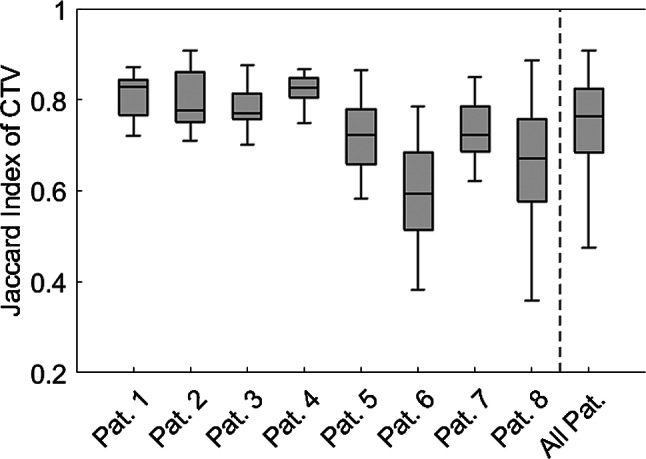
Jaccard index J of the CTV (bladder), related to the initial CTV in the pCT, for every individual patient as well as all patients merged

The Jaccard index J of the CTV had a median value of 0.76 [Q1: 0.68; Q3: 0.82] across all patients, with individual patient variation observed (e.g., Patient 1: median 0.83 [Q1: 0.77; Q3: 0.84]; Patient 6: median 0.59 [Q1: 0.51; Q3: 0.68]). Detailed data are presented in Fig. 3.

### Target dose differences

**Fig. 4:**
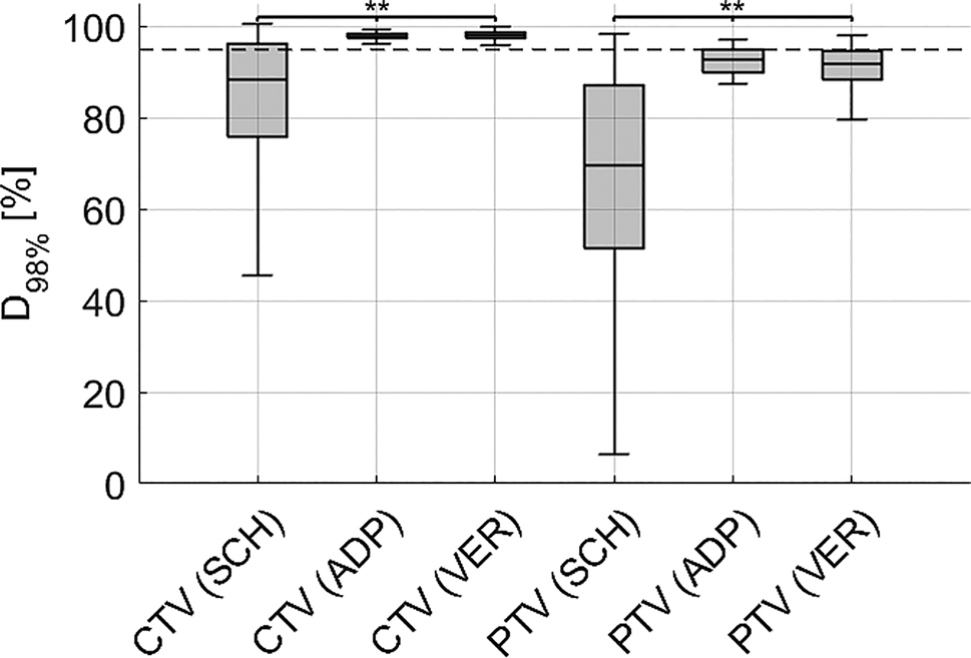
Dose coverage (D_98%_) for scheduled (SCH), adapted (ADP), and verification (VER) dose, for CTV (left) and PTV (right), merged for all patients. Internal clinical standard constraint (D_98%_ >95%) is marked by a dashed line. (**) indicates *p* < 0.05

The dose coverage for the CTV, assessed by the D_98%_ metric, showed a median value of 88.4% for SCH plans, 97.8% for ADP plans, and 98.1% for VER plans. For the PTV, the D_98%_ was 69.5% for SCH, 92.8% for ADP, and 91.8% for VER, respectively. Figure 4 visually presents these findings, highlighting significant differences in dose coverage across both CTV (SCH vs. ADP: *p* < 0.01; SCH vs. VER: *p* < 0.01) and PTV (SCH vs. ADP: *p* < 0.01; SCH vs. VER: *p* < 0.01). In addition, a reduction in the interquartile range (IQR) was observed when comparing SCH to ADP and SCH to VER. Individual patient-level results are provided in Supplement 1.

**Fig. 5:**
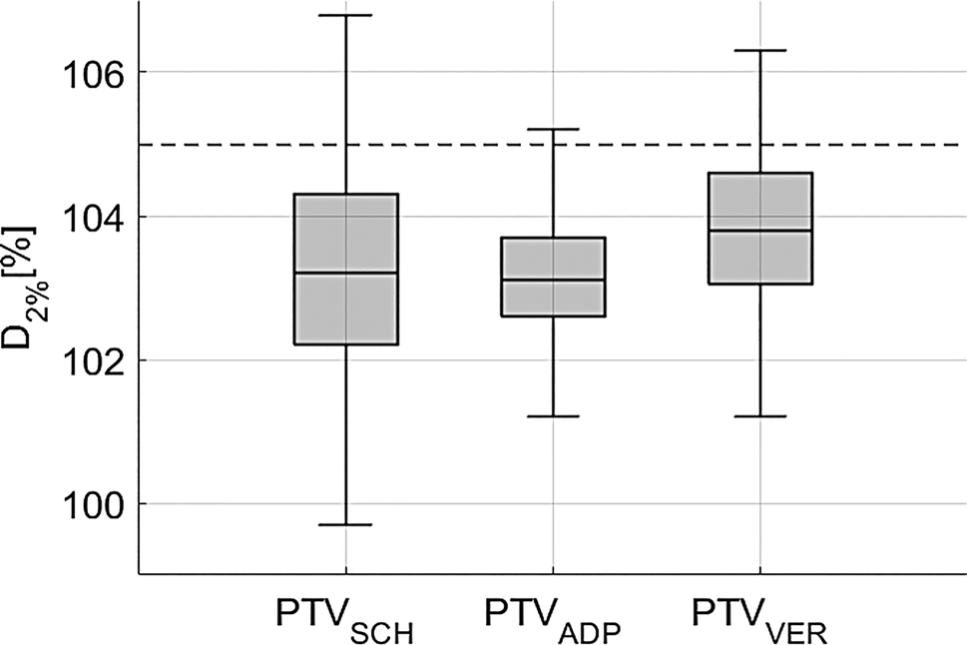
Dose near-max (D_2%_) for SCH, ADP and VER dose, for PTV, merged for all patients. Internal clinical standard constraint (D_2%_ ≤ 105%) is marked by a dashed line

For the PTV near-maximum dose, described by D_2%_, the difference between SCH and ADP was statistically not significant (median SCH: 103.2%; median ADP: 103.1%, *p* = 0.88). However, significant differences were observed between SCH and VER (median VER: 103.8%, *p* < 0.01) and between ADP and VER (*p* < 0.01). Figure 5 illustrates these dose differences, with the internal clinical standard constraint (D_2%_ ≤ 105%) marked by a dashed line. The IQR decreased from SCH to ADP and increased again to VER. Comprehensive statistical data on target doses are detailed in Table 2, individual patient-level results are provided in Supplement 2.

**Table 2:** The table presents statistical data for the CTV and PTV (D_98%_ and D_2%_) for SCH, ADP, and VER plans

	Para-meter	Plan	Median Dose [%/fx]	Q1 [%/fx]	Q3 [%/fx]	IQR	*p*-value (to SCH)	*p*-value (to ADP)
CTV	D_98%_	SCH	88.4	75.8	96.3	20.5		
		ADP	97.8	97.3	98.3	1.0	< 0.01	
		VER	98.1	97.5	98.7	1.2	< 0.01	< 0.01
PTV	D_98%_	SCH	69.5	51.3	87.0	35.7		
		ADP	92.8	89.7	94.8	5.1	< 0.01	
		VER	91.8	88.4	94.6	6.2	< 0.01	< 0.01
	D_2%_	SCH	103.2	102.2	104.3	2.1		
		ADP	103.1	102.6	103.7	1.1	0.88	
		VER	103.8	103.0	104.6	1.6	< 0.01	< 0.01

Median, first quartile (Q1), third quartile (Q3) interquartile range IQR (Q3-Q1) and p-values (Wilcoxon signed-rank test) are included

D_98%_>95% of the CTV and PTV and D_2%_<105% of the PTV (Table 1) were assessed for clinical acceptability. Based on these metrics, 19 out of 160 fractions (11.9%) in the SCH plans, 37 out of 160 fractions (23.1%) in the ADP plans, and 36 out of 160 fractions (22.5%) in the VER plans met this standard. For the initial treatment plan, 3 out of 8 patients fulfilled the criteria, while 5 out of 8 did not. These results indicate a significant improvement in plan acceptability when comparing SCH to ADP (McNemar test: OR 36.00, 95% CI [2.17-597.98], *p* < 0.01) and SCH to VER (McNemar test: OR 34.00, 95% CI [2.04-566.02], *p* < 0.01). It is important to note that a certain degree of target dose compromise was intentionally applied to protect the bowel, and this was incorporated into the optimization parameters, leading to a decrease in plan acceptability according to the above-mentioned criteria.

**Fig. 6:**
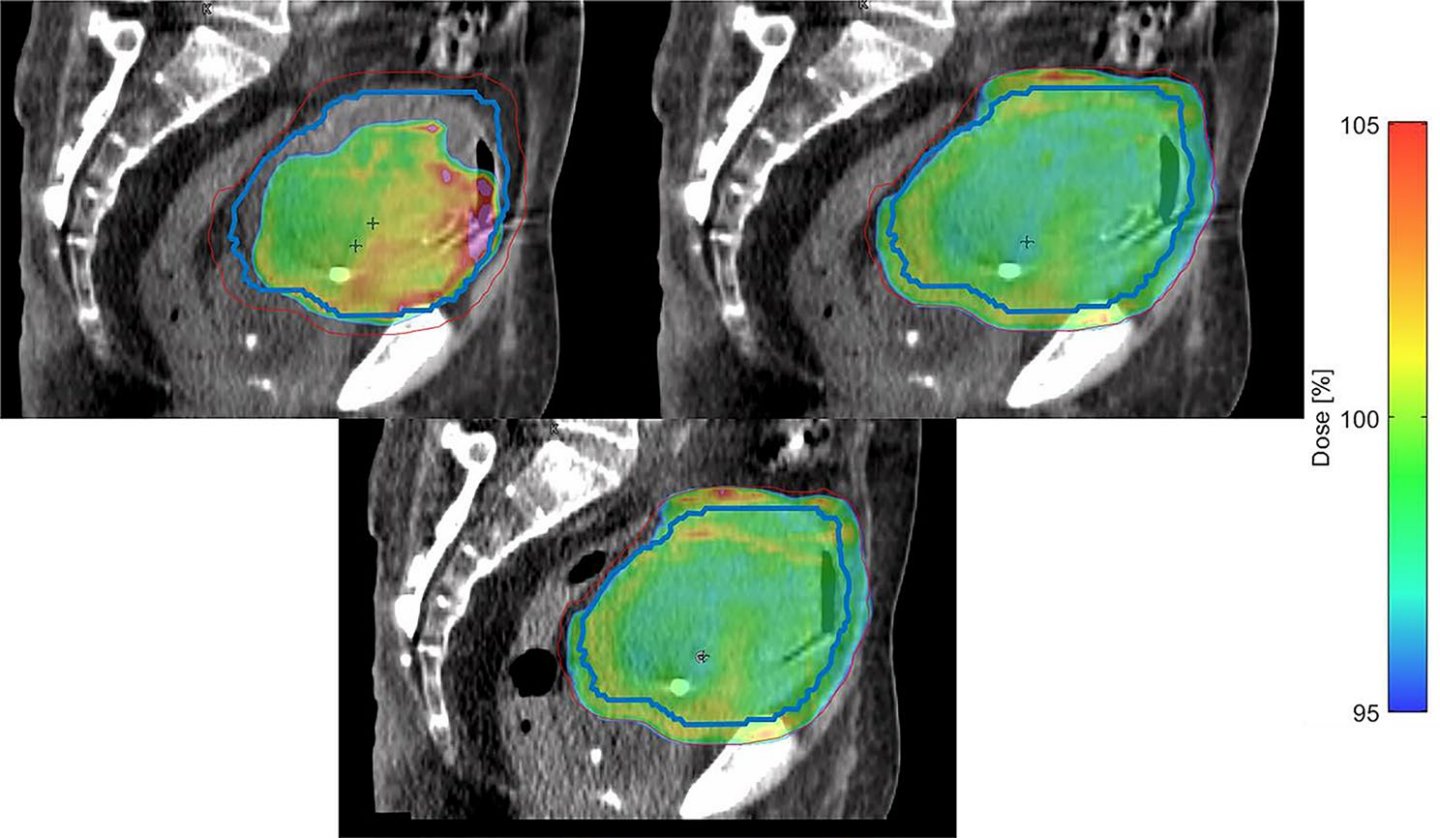
Visualization of the 95% isodose (blue) with a color wash up to 105% (red) of the prescribed dose. The scheduled plan (SCH, top-left) shows the CTV (blue) with insufficient coverage, likely due to bladder volume fluctuations. In contrast, the adapted plan (ADP, top-right) demonstrates an optimized dose distribution with improved target coverage. Bottom: Dose distribution reconstructed on the post-adaptive vCBCT (VER). These images show patient 8, fraction 7

Figure 6 depicts the effect of bladder volume variations on dose coverage for the CTV and PTV across the three plan types (SCH on the top-left, ADP on the top-right, and VER on the bottom). During this specific treatment session (patient 8, fraction 7), the bladder volume was approximately three times larger than observed in the initial pCT. The patient presented in this session with a markedly overfilled bladder and was evidently unable or unwilling to follow the prescribed voiding protocol. The figure showcases a treatment session where drinking and voiding instructions obviously could not be followed by the patient and a significantly increased bladder filling led to suboptimal dose distribution in the SCH plan, leaving portions of the CTV and PTV below the prescribed dose threshold. The ADP plan, however, demonstrates improved dose coverage, having been reoptimized to reflect the anatomy of the day. The VER plan reveals slight deviations from the ADP plan, reflecting potential intrafractional changes that occur during the process of adaptation.

### OAR dose differences

**Fig. 7:**
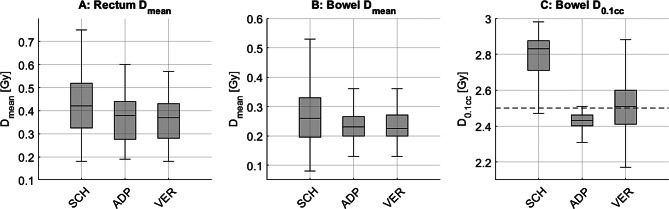
Dose exposure to rectum (**A**) and bowel (**B**) in terms of D_mean_ as well as bowel D_0.1cc_ (**C**) for SCH, ADP and VER dose. Internal clinical standard constraint for bowel D_0.1cc_ (≤ 2.5 Gy/fx) is marked by a dashed line

Figure 7 illustrates the dose exposure (measured as D_mean_) to the rectum (Fig. 7A) and bowel (Fig. 7B) across all patients for the SCH, ADP, and VER plans. A significant difference in D_mean_ for the bowel was observed when comparing SCH with both ADP and VER (*p* < 0.01 for both). Additionally, the IQR for bowel D_mean_ narrowed considerably, dropping from 0.13 for SCH to 0.06 for ADP and 0.07 for VER. In terms of rectal exposure, the D_mean_ showed significant reductions when comparing SCH with ADP and VER (*p* < 0.01 for both). The median rectal D_mean_ was 0.42 Gy/fx for SCH, 0.38 Gy/fx for ADP, and 0.37 Gy/fx for VER. Correspondingly, the IQR for rectal D_mean_ decreased from 0.19 for SCH to 0.16 for ADP and 0.15 for VER.

For bowel D_0.1cc_, significant differences were found between SCH and ADP, as well as between SCH and VER (*p* < 0.01 for both). The median D_0.1cc_ was 2.83 Gy/fx for SCH, compared to 2.43 Gy/fx for ADP and 2.51 Gy/fx for VER. The IQR for bowel D_0.1cc_ decreased from 0.15 for SCH to 0.06 for ADP, but increased to 0.19 for VER, as shown in Fig. 7C. In addition, bowel Dmean was 0.26 Gy/fx for SCH, compared to 0.23 Gy/fx for ADP and 0.23 Gy/fx for VER, with significant differences between SCH and ADP as well as between SCH and VER (*p* < 0.01 for both). The clinical standard constraint D_0.1cc_ ≤ 50 Gy (resp. ≤ 2.5 Gy/fx) is marked by a dashed line.

Respective statistical data are detailed in Table 3. Individual patient-level results are provided in Supplement 3–5.

**Table 3:** Statistical data for the rectum and bowel D_mean_ as well as bowel D_0.1cc_ for SCH, ADP, and VER plans

	Para-meter	Plan	Median Dose [Gy/fx]	Q1 [Gy/fx]	Q3 [Gy/fx]	IQR	*p*-value (to SCH)	*p*-value (to ADP)
Rectum	D_mean_	SCH	0.42	0.33	0.52	0.19		
		ADP	0.38	0.28	0.44	0.16	< 0.01	
		VER	0.37	0.28	0.43	0.15	< 0.01	0.20
Bowel	D_mean_	SCH	0.26	0.20	0.33	0.13		
		ADP	0.23	0.20	0.26	0.06	< 0.01	
		VER	0.23	0.20	0.27	0.07	< 0.01	0.15
	D_0.1 cc_	SCH	2.83	2.72	2.87	0.15		
		ADP	2.43	2.40	2.46	0.06	< 0.01	
		VER	2.51	2.41	2.60	0.19	< 0.01	< 0.01

Median, first quartile (Q1), third quartile (Q3) interquartile range IQR (Q3-Q1) and p-values (Wilcoxon signed-rank test) are included

### Target surface dose

The surface dose analysis of the targets focused on points on the CTV surface falling below the threshold value of 90% of the prescribed dose. A comparative assessment of SCH and ADP plans revealed a reduction in the percentage of surface points failing to meet the threshold. Specifically, SCH plans exhibited a total of 48.3% of points falling below the threshold, while ADP plans showed a reduction to 38.9%, underlining an improvement in dose coverage with ADP. For the eight initial treatment plans, the corresponding failure rate was 36.4%.

**Fig. 8:**
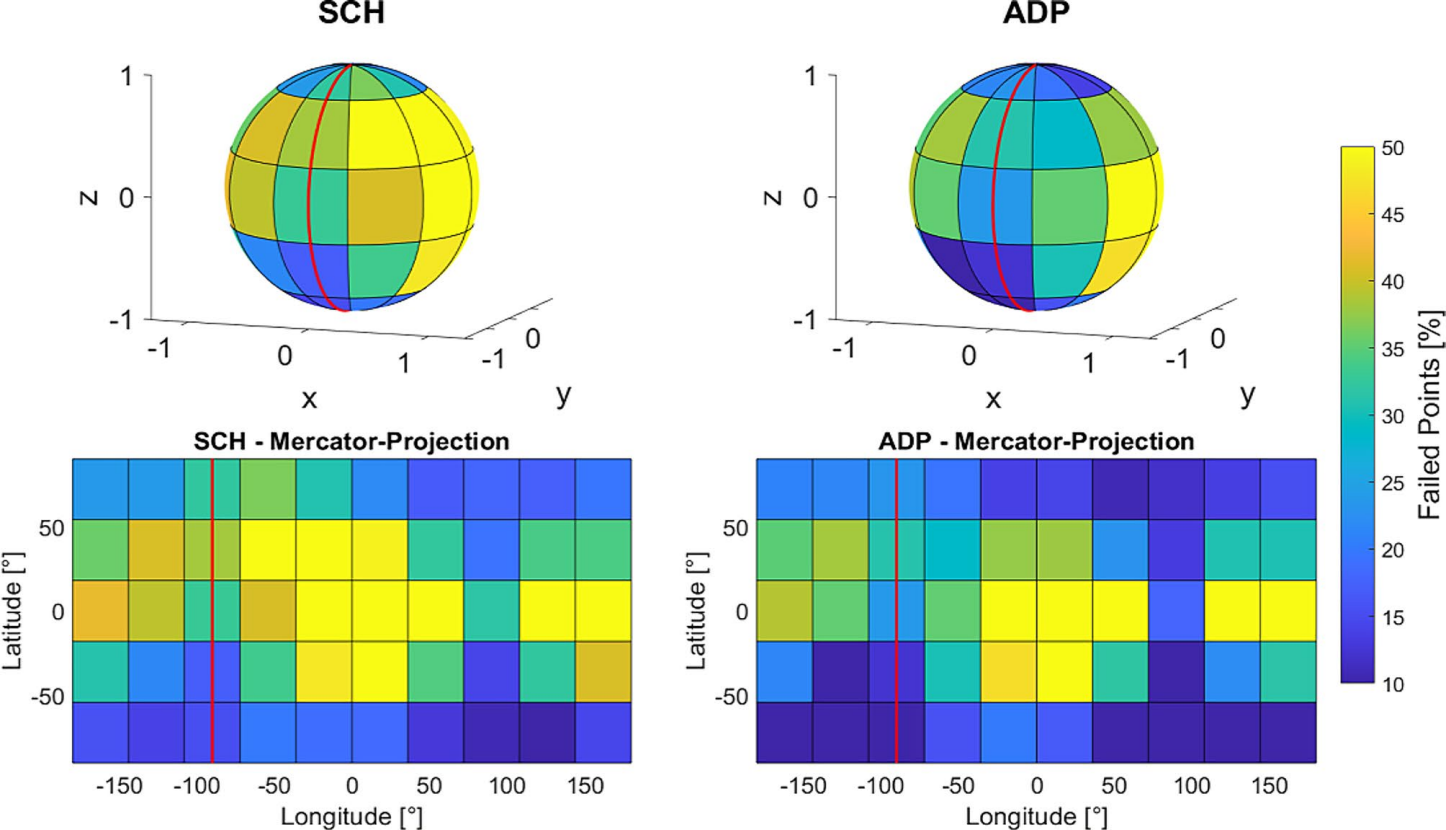
Failed CTV surface points (< 90% of prescribed dose) per segment for SCH (left) and ADP (right) plans of all patients. The spherical representation is shown at the top, with the Mercator projection below. A red line indicates the anterior direction (head to feet) to aid orientation

**Fig. 9:**
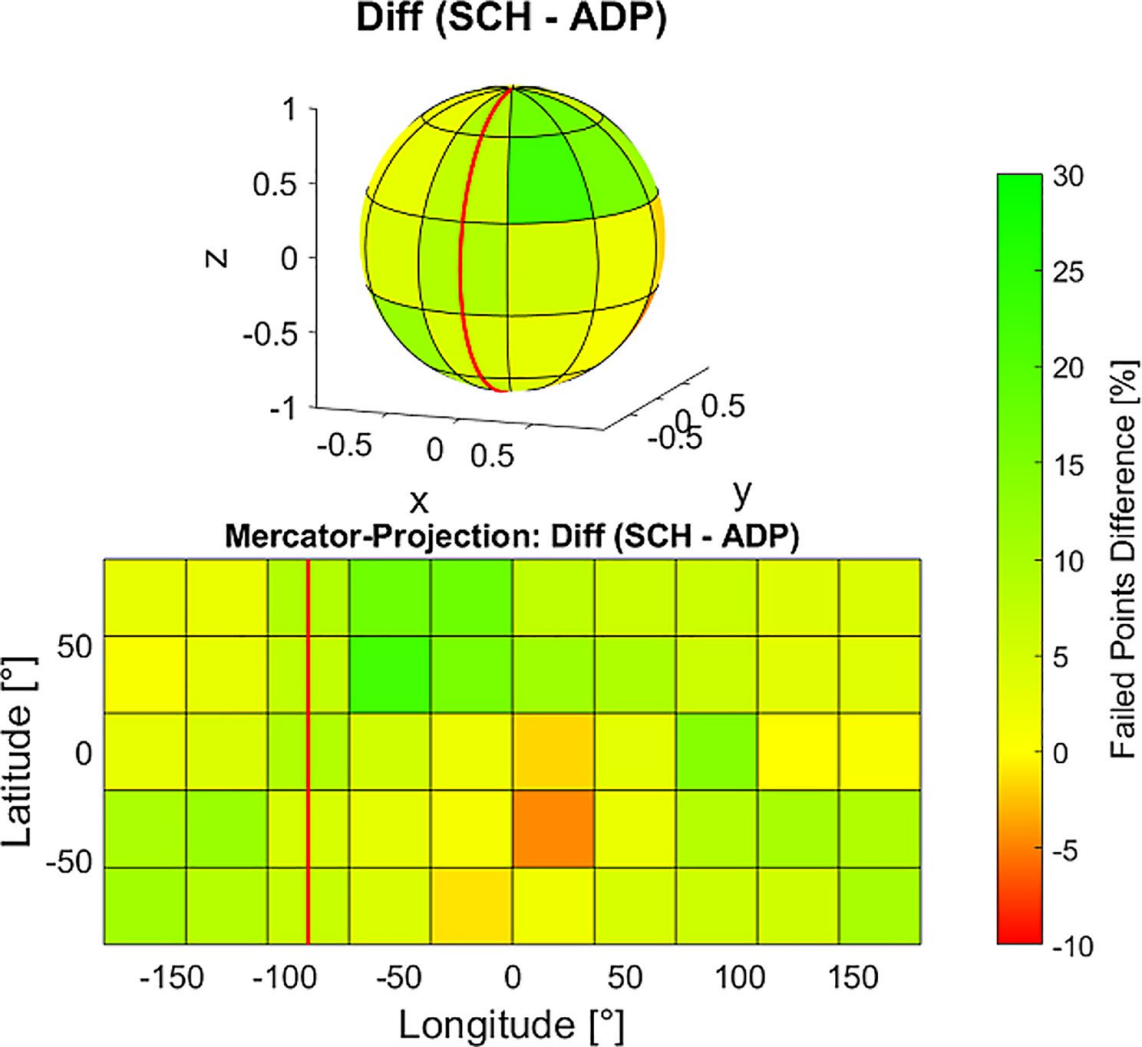
Difference in failed CTV surface points (< 90% of prescribed dose) per segment (SCH – ADP) of all patients. Negative values indicate a degradation in mean surface dose for the respective segment. The spherical representation is shown at the top, with the Mercator projection below. A red line indicates the anterior direction (head to feet) to aid orientation

Figure 8 illustrates the spatial distribution of sub-threshold points, segmented into latitudinal bands and longitudinal sectors on a unit sphere centered at the CTV’s center of mass, averaged across all 160 fractions analyzed. To present data from all directions of the sphere, a Mercator projection was used, mapping all information into a two-dimensional plane. Figure 9 shows the fraction-wise difference between SCH and ADP per segment. This spatial mapping highlights that failure points predominantly occurred in the posterior region for both SCH and ADP plans. However, ADP plans show a noticeable reduction in failures across most regions, as can also be seen in the difference plot. Nevertheless, an elevated level of failures in ADP persists in the left posterior area compared to other regions, a feasible reason for this will be addressed in the discussion section.

**Fig. 10:**
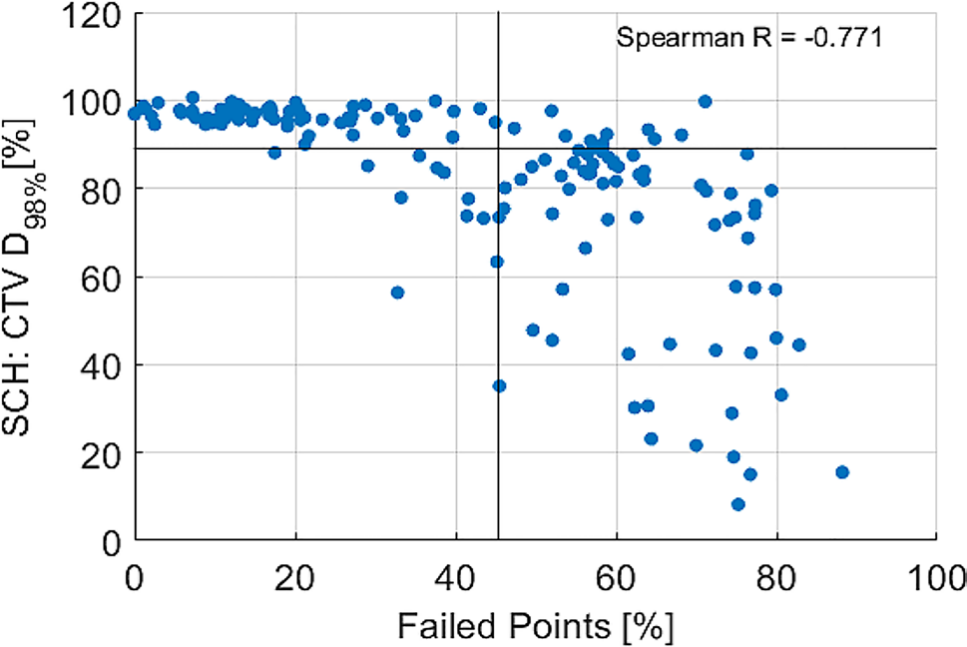
Scatter plot for CTV coverage (SCH) D_98%_ [%] against surface failed points [%], Spearman *R* = -0.771. Horizontal and vertical lines represent the specific median

Figure 10 illustrates the relationship between the percentage of failed surface points and the dose coverage of the CTV in terms of D_98%_, indicating a weak correlation between the two variables (Spearman *R* = -0.771).

### Treatment time

Adaptive treatments were significantly longer than IGRT treatments (*p* < 0.01).The total treatment time for IGRT was median 9 min [Q1: 8; Q3: 12], the time required for oART across all fractions in this study had a median of 14 min [Q1: 12; Q3: 16] with the initial treatment of each patient lasting median 16 min [Q1: 12; Q3: 21].

## Discussion

### Patient selection and plan generation

The selection of patients for this study was primarily determined by the availability of open slots at the linear accelerator. This approach facilitated flexibility in patient inclusion while optimizing the use of treatment resources. Further developments by the vendor, particularly improvements in AI processing and the efficiency of optimization algorithms, could enhance overall clinical workflow and potentially broaden the practical applicability of VMAT as a more complex and faster delivery technique.

### Geometrical differences

Even though patients were carefully instructed about drinking and urinating, there was considerable variation in bladder (CTV) volume across treatment sessions in all eight cases, which was effectively demonstrated using oART. Although many efforts have been made to maintain consistent organ volumes [29], such variability has been previously described for bladder treatment [11] as well as for other pelvic sites, including the prostate [30–32], prostate bed [20] and cervix [33]. This indicates that many patients have difficulty adhering to fluid intake and voiding protocols. While some degree of natural variation in bladder filling is to be expected, it alone cannot account for the extent of the observed inter- and intra-patient variability. Patient-individualized drinking and voiding regimens may help improve bladder volume reproducibility but are often time-consuming in practice and yield inconsistent results. Adaptive techniques may solve these problems [34]. Moreover, with oART, time-consuming trips to the toilet and repeated imaging or repositioning procedures can often be avoided, further streamlining the workflow and improving patient comfort. Invasive or device-based strategies to standardize bladder filling such as catheterization [9] or ultrasound-assisted volume control [7, 8] have been proposed as alternatives. However, these approaches are typically associated with increased procedural effort, patient discomfort, and limited reliability in clinical routine. Their use may be considered in settings where adaptive radiotherapy is not available.

Furthermore, the Jaccard index, which measures geometric similarity, showed considerable variation. However, since most commonly used treatment planning systems do not routinely report the Jaccard index or the closely related DICE index, an alternative metric is required. The relative bladder volume - derived from the absolute volume in relation to the bladder’s initial planning volume - offers a simpler and more widely applicable surrogate for tracking these variations. In this cohort, relative bladder volume deviated from unity in all patients, with higher-than-planned volumes in five cases and lower volumes in three. Interpatient variability also differed markedly, with broader IQRs observed in some patients (e.g., patients 6 and 8) compared to others (e.g., patients 1 and 4). The relative volume calculated for the total cohort was greater than unity, primarily influenced by large deviations in patients 5, 6, and 8. Possible reasons for these deviations could include patient nervousness during pCT and improved compliance with voiding instructions as treatment progressed. However, such patterns were only observed in five of the eight patients, limiting generalizability. Due to the limited sample size, no generalizable trend regarding the direction of deviation can be concluded.

In the context of oART with the Ethos system, daily plan calculation is based on sCT generated by deformable registration of the planning CT to the daily CBCT. This sCT is hidden from the user and used solely for dose calculation. As a result, the accuracy of the deformation and its potential impact on dose calculation remain unclear. Other studies, particularly in patients with significant weight loss [35], have suggested that deformable image registration used for sCT creation may introduce dosimetric uncertainties. This aspect warrants further investigation, especially for cases with pronounced anatomical variation.

### Target and organs at risk dose differences

For the target volumes, oART significantly improved dose coverage from SCH to ADP and to VER for both CTV and PTV. This is consistent with findings from other studies [25] and also for other entities [19, 20]. Although there was a slight decrease in coverage from ADP to VER for PTV, the dose remained clinically acceptable and continued to outperform SCH, demonstrating the sustained advantage of oART in target dose coverage. Interestingly, for the CTV, D_98%_ increased slightly but significantly from ADP to VER. This counterintuitive finding was considered incidental and clinically irrelevant, as absolute changes were minimal and did not affect the overall interpretation of target coverage quality. Furthermore, the IQR for dose coverage decreased significantly when comparing SCH to ADP and VER, highlighting the value of oART in improving consistency and reproducibility of target dose coverage. This improvement likely remains valid even under the uncertainties potentially induced by intraprocedural motion, possibly indicated by VER. It should be noted, however, that for some patients (e.g., patients 1–3), the gain in target coverage was minimal, though still positive. In these cases, the benefit of oART was more pronounced in terms of bowel sparing, as illustrated by notable reductions in D_0.1cc_ dose exposure. Detailed individual results are provided in Supplement 1 and 5. For D_2%_, statistically significant differences were observed between SCH and ADP as well as between SCH and VER, although these differences are likely not clinically meaningful. Overall plan acceptability improved significantly from SCH to ADP and VER, although the overall number of acceptable plans remained modest, as target dose compromises were deliberately applied to prioritize bowel protection. Even the initial treatment plans met the acceptability criteria in only 3 out of 8 cases.

For OAR, metrics such as D_mean_ for both bowel and rectum showed improvements with ADP and VER compared to SCH, though no significant differences between ADP and VER were found. However, both the absolute D_mean_ values and hence the differences observed between SCH, ADP, and VER were very low, and are therefore unlikely to be of clinical relevance. The reduced IQR in these parameters suggests a degree of robustness with respect to intraprocedural variation and possibly also intrafractional effects, although the latter was not directly assessed in this study. This supports the potential of oART to enhance reproducible dose sparing for OARs.

In contrast, parameters reflecting near-maximum exposure, such as bowel D_0.1cc_, showed significant reductions with ADP but increased again in VER. This indicates that such highly localized dose metrics are particularly sensitive to anatomical changes that may occur interfractional or even during the time between adaptation and delivery. Therefore, they depend strongly on accurate daily imaging and rapid plan execution or even online adaptation [36]. Additionally, the speed of the adaptation workflow becomes critical, as the absence of intrafractional motion management limits the ability to fully mitigate these variations. Moreover, commercially available systems for intrafractional motion management, if available, merely rely on techniques such as X-rays or surface tracking, which are incapable of monitoring abdominal soft tissue movement.

### Target surface dose

A novel approach to evaluate spatial distribution of dose coverage to target volumes was provided. It was found that surface dose coverage improved overall with ADP compared to SCH, as evidenced by a lower percentage of total surface points failing to meet the 90% prescription dose threshold. However, the posterior region consistently showed higher failure rates even with ADP. This can be attributed to the strict dose constraints (D_0.1cc_) applied to protect the bowel, which is in close proximity to the target in this region. Consequently, the locally higher failure rates highlight the intended underdosage of the target volume in this area.

Commonly used DVH parameters, such as D_98%_ or V_95%_, describe the dose to the overall target volume. In the case of the bladder, this volume predominantly consists of urine and therefore the analysis of target surface dose may be more relevant for bladder radiotherapy. The tumorous tissue is generally located within the bladder wall, making it adjacent to the structure’s surface and challenging to target accurately due to the bladder’s dynamic nature. The presented spatial surface analysis may provide an important complement to conventional DVH-based evaluation, as it enables the detection of localized, near-surface underdosage that may be underestimated or entirely missed by volumetric metrics such as D_98%_, particularly in larger targets. This is further supported by the observed weak Spearman correlation between the percentage of failed surface points and D_98%_, indicating that cases with substantial peripheral underdosage may still show high overall dose coverage according to standard DVH parameters. Hence, relying solely on D_98%_ may obscure clinically relevant underdosage near the bladder wall. The derivation of anisotropic PTV margins, as already described in offline adaptive settings [37], may offer a viable strategy to address this limitation by compensating for localized variations in coverage without compromising OAR protection.

It should also be noted that the failure rates reported in this work may appear relatively high. However, as this surface-based evaluation method represents a novel approach to dose assessment, the observed values might reflect a shift in interpretive standards that will require future contextualization. Additionally, localized dose drops near the target surface are inherently more likely than widespread volumetric underdosage, which makes the detection of a larger number of failing surface points a plausible and expected outcome. This interpretation is further supported by the analysis of the initial treatment plans on the pCT, which showed a failure rate comparable to that observed for ADP. This suggests that the suboptimal surface coverage is not merely a product of daily anatomical variation or replanning, but is already present in the original optimization and likely reflects clinically accepted standards.

### Treatment time

Adaptation in radiotherapy inevitably increases treatment time. In CBCT-based prostate bed treatments, for example, adaptation times can extend beyond one hour [20], highlighting the significant time demand of the process. However, in bladder cancer treatments, workflows were notably faster, as the bladder - serving as both an organ and the CTV - could be efficiently auto-contoured and propagated to define the CTV and PTV. As a result, the median treatment time for oART was only 1.5 times longer than for conventional IGRT, with less pronounced variability compared to other adaptive sites [20]. This demonstrates that while oART for bladder cancer remains time-intensive relative to IGRT, it is considerably more efficient within the broader context of adaptive radiotherapy.

When comparing CBCT-based with MRI-based oART systems [38], several key differences should be considered. CBCT-based oART generally enables faster adaptation workflows, which is particularly advantageous for sites like the bladder, where rapid replanning helps mitigate intrafractional changes. However, MRI-based systems offer superior soft tissue contrast, which can improve target and OAR delineation in anatomically complex regions. Moreover, MRI-based oART platforms can provide continuous or 2D real-time imaging to monitor intrafractional motion, an option not available with current CBCT-based solutions. Therefore, the success of CBCT-based oART relies on achieving sufficient image quality and minimizing the time between imaging and treatment delivery to reduce the risk of anatomical variation. In addition, CBCT-based oART systems are generally less costly and more widely accessible than MRI-based solutions, which may facilitate broader implementation in routine clinical practice.

### Limitations

Some limitations of this study should be acknowledged. As a single-institution analysis with a limited number of patients, the generalizability of the results may be constrained, despite the relatively high number of evaluated fractions. Additionally, no CBCT imaging was performed after radiation delivery (post-RT-CBCT), which limits the ability to assess potential intrafractional anatomical changes occurring beyond the adaptation and verification steps. This decision was made deliberately to avoid unnecessary imaging dose in consideration of radiation protection principles. While the adaptive workflow followed a standardized protocol, variations in physician involvement across treatment fractions may have introduced intra- and inter-observer variability in contour review and plan selection.

Moreover, the long-term clinical impact of oART remains uncertain, as prospective data on outcomes such as tumor control or late toxicity are still lacking.

## Conclusion

CBCT-based online adaptive radiotherapy for bladder cancer demonstrates significant improvements in target dose coverage and reduces variability in organ-at-risk sparing compared to non-adaptive IGRT approaches. The adaptive workflow effectively accommodates daily anatomical variations, allowing for intentional under-dosage in areas adjacent to sensitive and highly mobile organs. A novel method to analyze the spatial surface dose coverage of targets highlighted the advantages of ADP over SCH and may serve as a tool to evaluate anisotropic margins tailored to adaptive treatments in the future.

However, these benefits come with increased complexity in treatment process, extended durations, and higher labor demands, raising concerns about clinical feasibility. While these challenges must be addressed through continued workflow optimization, it is important to consider the potential long-term benefits. By reducing toxicity and minimizing the risk of recurrence, oART could ultimately lead to cost savings and improved patient outcomes. Given that it provides the best possible treatment solution for patients, there is a strong imperative to overcome the associated commercial and organizational hurdles and facilitate its broader integration into routine clinical practice. Nevertheless, the long-term clinical outcomes of oART remain to be confirmed in prospective studies.

## Supplementary Information

Below is the link to the electronic supplementary material.

Supplementary Material 1

## Data Availability

No datasets were generated or analysed during the current study.

## Abbreviations

ADP: Adapted Plan
ALARA: As low as reasonably achievable
CBCT: Cone Beam Computed Tomography
CI: Confidence Interval
CTV: Clinical Target Volume
DICOM: Digital Imaging and Communications in Medicine
DVH: Dose-Volume-Histogram
fx: fraction
HU: Hounsfield Units
IGRT: Image Guided Radiotherapy
IMRT: Intensity Modulated Radiotherapy
IoU: Intersection over Union
IQR: Interquartile Range
MLC: Multi Leaf Collimator
MU: Monitor Unit
OAR: Organs at Risk
oART: online Adaptive Radiotherapy
OR: Odds Ratio
pCT: Planning Computed Tomography
PTV: Planning Target Volume
Q1: 1st quartile
Q3: 3rd quartile
SCH: Scheduled Plan
sCT: synthetic Computed Tomography
vCBCT: verification Cone Beam Computed Tomography
VER: Verification Plan
VMAT: Volumetric Modulated Arc Therapy
