# Supplementary material for: CBCT-based online adaptive radiotherapy of the bladder – geometrical and dosimetrical considerations compared to conventional IGRT
Source: Radiat Oncol. 2025 Aug 14;20:128. doi: 10.1186/s13014-025-02710-y (PMC12351964; doi:10.1186/s13014-025-02710-y)
Supplement: Supplementary file 1 — Supplementary Material 1 [file 13014_2025_2710_MOESM1_ESM.docx]

### Supplement 1


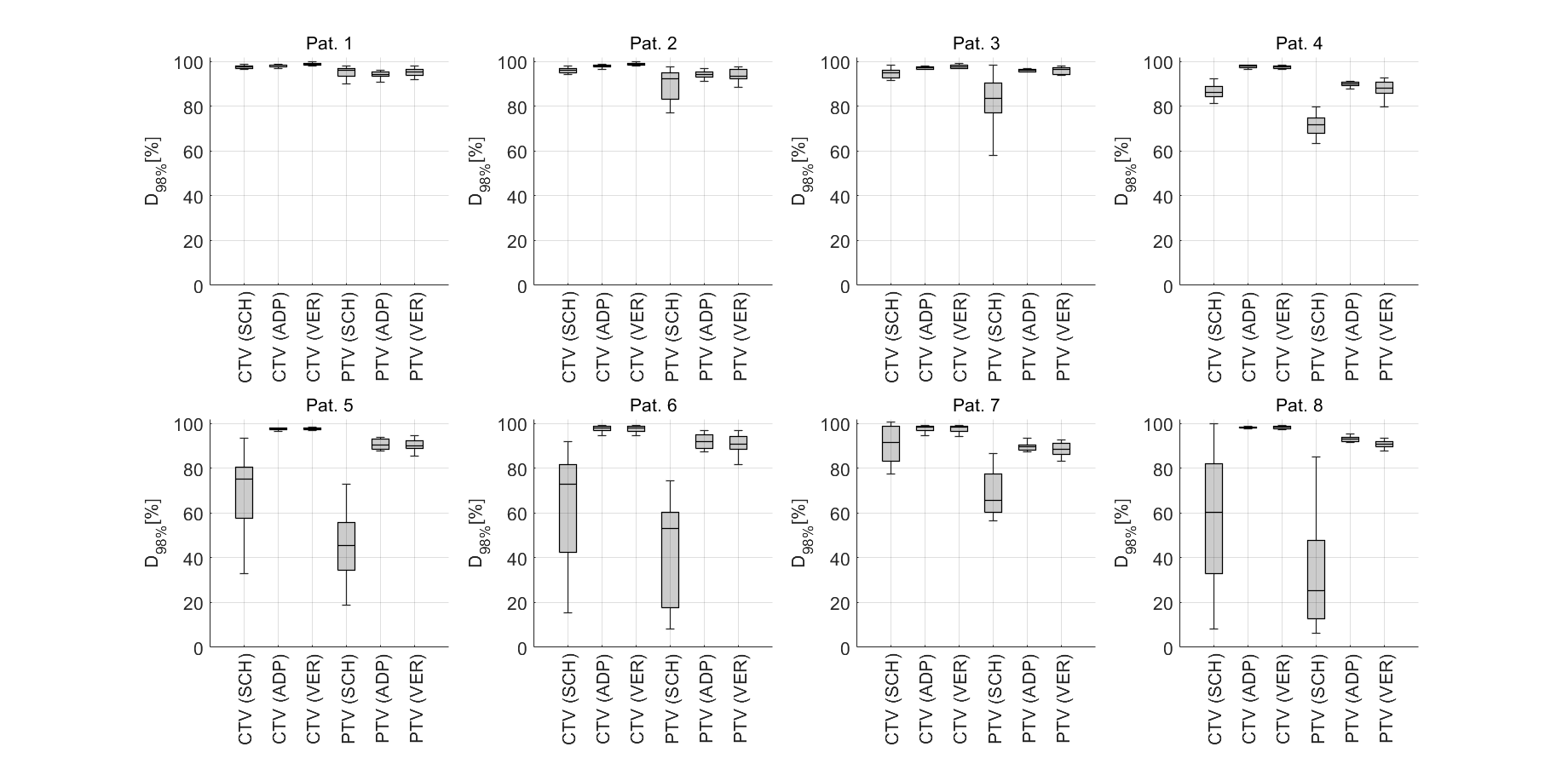


Figure 1 Dose coverage (D_98%_) for scheduled (SCH), adapted (ADP), and veriﬁcation (VER) dose, for CTV (left) and PTV (right), for all individual patients.

### Supplement 2


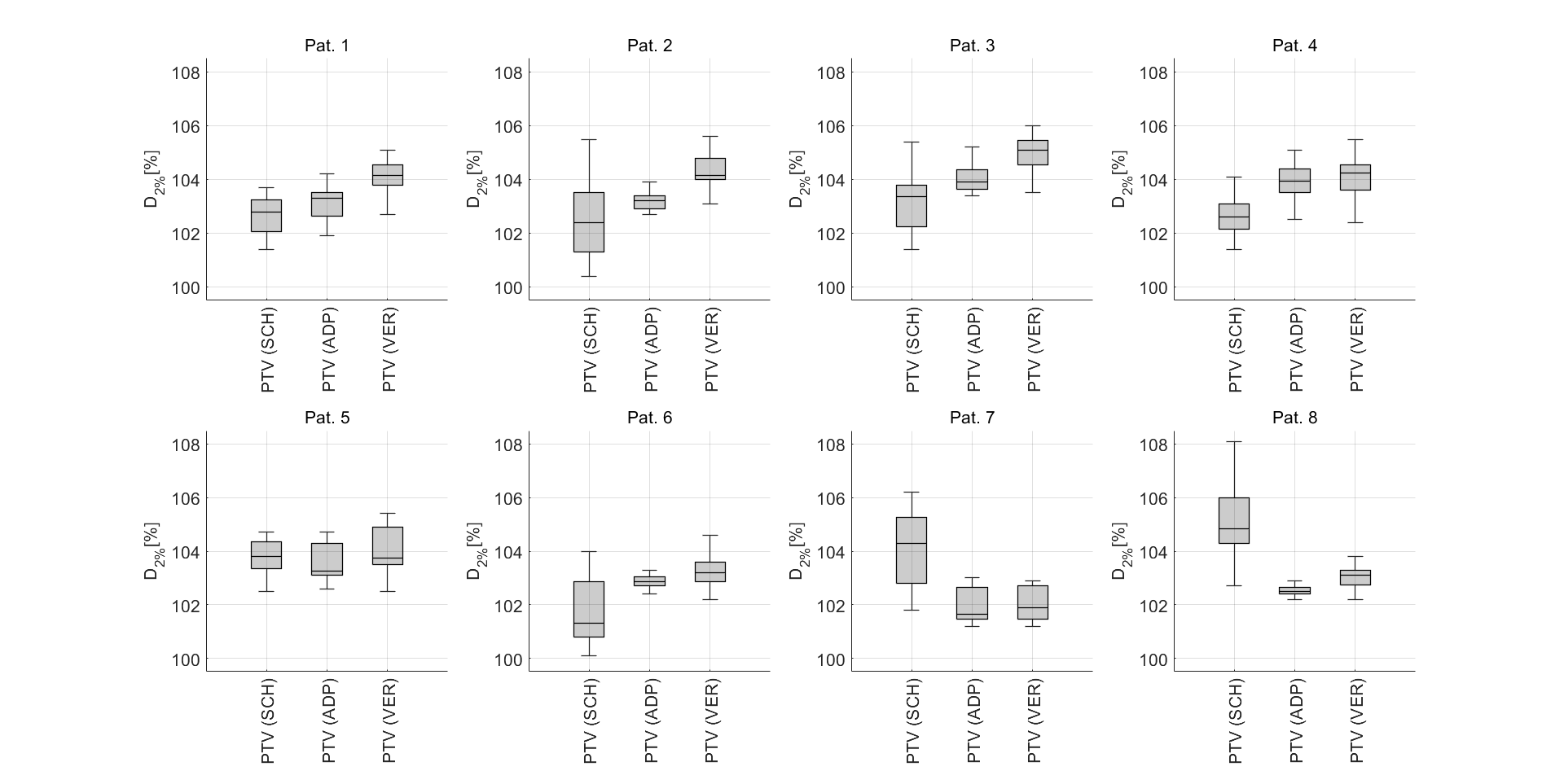


Figure 2 Dose near-max (D_2%_) for SCH, ADP and VER dose, for PTV, for all individual patients.

### Supplement 3


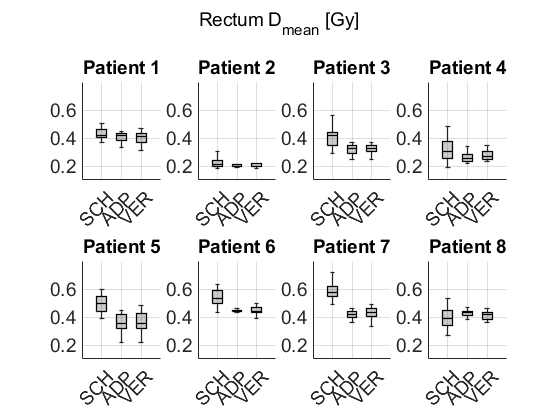


Figure 3 D_mean_ to rectum for SCH, ADP and VER dose, for all individual patients.

### Supplement 4


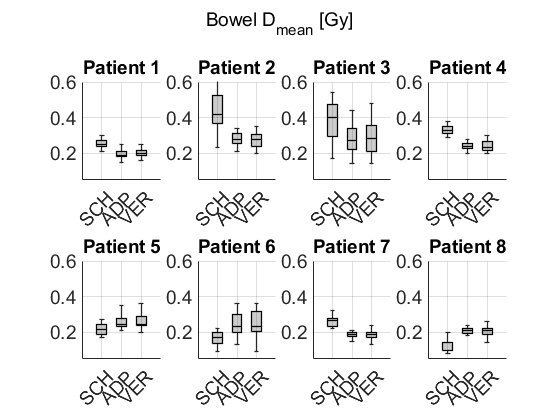


Figure 4 Dmean to bowel for SCH, ADP and VER dose, for all individual patients.

### Supplement 5


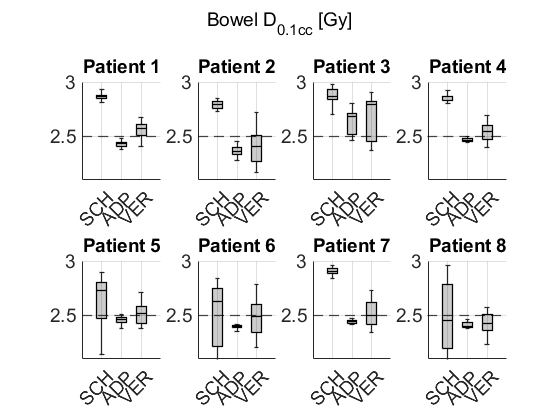


Figure 5 D_0.1cc_ to bowel for SCH, ADP and VER dose, for all individual patients.
